# Functional outcomes and quality of life after surgical treatment of spinopelvic dissociation: a case series with one-year follow-up

**DOI:** 10.1186/s12891-021-04676-w

**Published:** 2021-09-15

**Authors:** Giedrius Petryla, Rokas Bobina, Valentinas Uvarovas, Jaunius Kurtinaitis, Tomas Sveikata, Sigitas Ryliškis, Giedrius Kvederas, Igoris Šatkauskas

**Affiliations:** Clinic of Rheumatology, Orthopaedics Traumatology and Reconstructive Surgery, Faculty of Medicine, Vilnius University, Vilnius, Lithuania

**Keywords:** Spinopelvic dissociation, Quality of life, Pelvic function, Sacral fracture

## Abstract

**Background:**

Spinopelvic dissociation is a transverse sacral fracture in conjunction with a vertical fracture of the sacrum on both sides, which causes the dissociation of the upper sacrum and spine from the pelvis. The most common causes of these fractures are high energy injuries such as falls from height or motor vehicle accidents. Spinopelvic dissociation is rare and heterogenous with severe associated injuries. The aim of this study was to assess the injury characteristics, changes in the quality of life and functional outcomes in the 1-year period after spinopelvic dissociation.

**Materials and methods:**

During the period of 4 years (January 2016 and January 2020), 17 patients with spinopelvic dissociation were admitted to our centre and included in this single-centre prospective cohort study. One patient died during the admission; therefore 16 patients were enrolled in the analysis. Patients were followed-up for 12 months. The quality of life changes were evaluated via the SF-36 questionnaire, and the functional outcomes were evaluated using the Majeed pelvic score. Patients completed their questionnaires twice: firstly during hospitalization (regarding their pre-traumatic condition); and once again 1 year after their injury (regarding their current condition).

**Results:**

The mean age of the patients was 40.2 ± 17.7 years. Mean Majeed, PCS and MCS scores of SF-36 before the injury were 95.81 ± 9.50, 55.87 ± 8.89, and 43.76 ± 12.45, respectively. Mean Majeed, PCS and MCS scores 1 year after the injury were 71.13 ± 20.98, 43.45 ± 9.64, and 43.41 ± 7.56. During the period of 1 year after the injury, Majeed and PCS results reduced statistically significantly (*P* = 0.001 and *P* = 0.003, respectively), while MCS results remained similar (*P* = 0.501).

**Conclusions:**

According to the data of our study, for patients with spinopelvic dissociation functional outcomes are significantly reduced and only one-third of the patients achieved pre-traumatic functional outcomes 1 year after the injury.

## Introduction

Spinopelvic dissociation is a transverse sacral fracture in conjunction with a vertical fracture of the sacrum on both sides, which causes the dissociation of the upper sacrum and spine from the pelvis [1–3]. Spinopelvic dissociation is uncommon injury—accounting for approximately 3–5 % of all sacral fractures [4, 5]—but carry a high rate of neurological injuries affecting bowel, bladder, and lower extremity function [3]. Traumatic spinopelvic dissociations most commonly occur due to high energy trauma such as falls from height or motor vehicle accidents [3, 6, 7]. Roy-Camille describes them as the “suicidal jumper’s fracture,” because one common mechanism of injury is a jump from height during a suicide attempt [8]. These injuries are associated with local soft tissue damage, sacral nerve root damage, multiple other visceral organ injuries, other bone fractures, and hemorrhages [1, 3, 9].

To predict the neurological deficit and functional outcomes, two classification systems are frequently used for spinopelvic dissociation: Roy-Camille classification, which was later modified by Strange-Vognsen and Lebech, and Denis classification [2, 8, 10].

There are few studies, with small cohorts, that have reported on the quality of life and functional outcomes of patients after spinopelvic dissociation [11–13]. This indicates that spinopelvic dissociation is a rare injury. In addition, functional outcomes and quality of life are difficult to assess due to associated injuries and high mortality rates [3]. The wider literature suggests that neurological impairment is related to poor functional outcomes and quality of life after spinopelvic dissociation, influencing the dysfunction of the bladder, bowel, and lower limbs [4].

In the present study, we evaluated the quality of life and functional outcomes of patients with spinopelvic dissociation using the SF-36 and Majeed pelvic score instruments. The aim of this study was to assess the injury characteristics, changes in the quality of life and functional outcomes in the 1-year period after spinopelvic dissociation. Our hypothesis was that patients who experience spinopelvic dissociation do not return to baseline functional and quality of life levels within a 1-year period.

## Materials and methods

This study was a single-centre prospective cohort study that included patients with spinopelvic dissociation after high energy injury admitted to a single trauma centre during the period of 4 years (January 2016 – January 2020). During this period, 17 (3.8 %) patients with spinopelvic dissociation—out of a total of 418 patients with pelvic fractures—were admitted to our centre. One patient died at the time of admission and was excluded from the research. In addition, patients with pathological or osteoporotic sacral fractures, pregnant women, and patients with mental illnesses were not included in the research.

All of the patients were treated surgically. The study was approved by the Vilnius Regional Biomedical Research Ethics Committee (approval No. 158200-16-868-394, and informed consent was obtained from every participant.

Pelvic computed tomography (CT) was performed for each patient for a more accurate diagnosis and view of the fracture pattern. Sacral fractures were classified according to the Roy-Camille and Denis classifications using CT images. Out of 16 patients, 12 sustained U-shaped, 3 – H-shaped, and 1 – Lambda-shaped sacral fracture.

The personal data of each patient was collected, which included: gender; age; comorbidities; injury severity score (ISS); concomitant injuries; surgical or non-surgical treatment; other surgery; length of surgery; length of hospital stay; adverse events; complications; and mortality.

The physical component summary (PCS) and mental component summary (MCS) were evaluated via the SF-36 questionnaire [14], and the functional outcomes were evaluated using the Majeed pelvic score. These functional results were then graded as follows: ≥85 excellent; 70–84 good; 55–69 fair; <55 poor [15]. Patients completed their questionnaires twice: firstly during hospitalization (regarding their pre-traumatic condition); and once again 1 year after their injury (regarding their current condition).

**Fig. 1:**
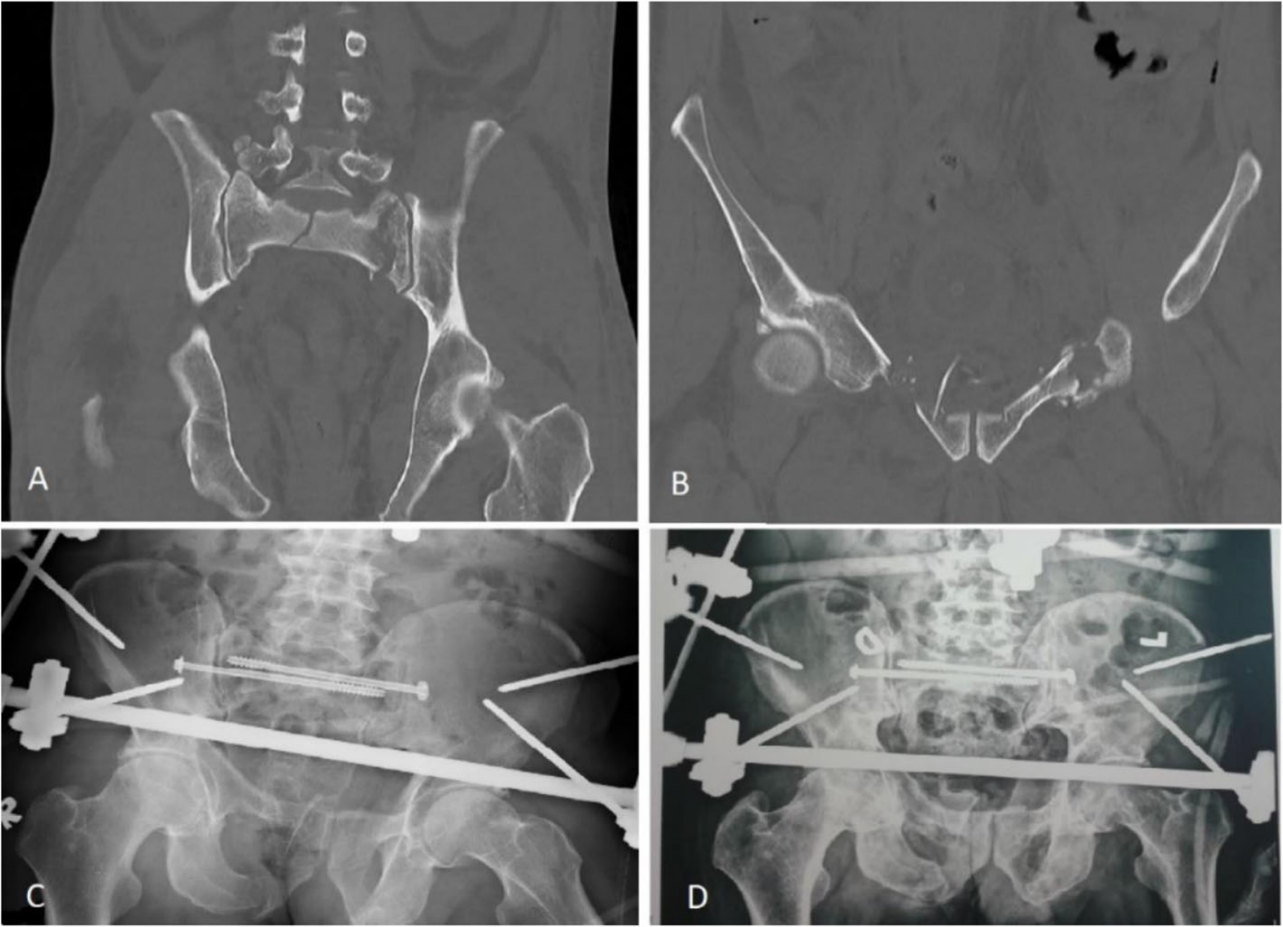
**A-D** Sacroiliac screw fixation in combination with external fixation of the anterior pelvic ring

No formal surgery protocol for spinopelvic dissociation was used. The choice of fracture fixation, approaches and surgery technique depended on the displacement of the fracture, stability of the pelvic ring, other visceral organs injuries and the attending surgeon’s preference. All surgeries were performed by the same team consisting of two experienced orthopaedic surgeons. An anterior definitive external fixation in combination with percutaneous screw fixation of the posterior pelvic ring was applied to one patient with severe urinary bladder injury (Fig. 1).

**Fig. 2:**
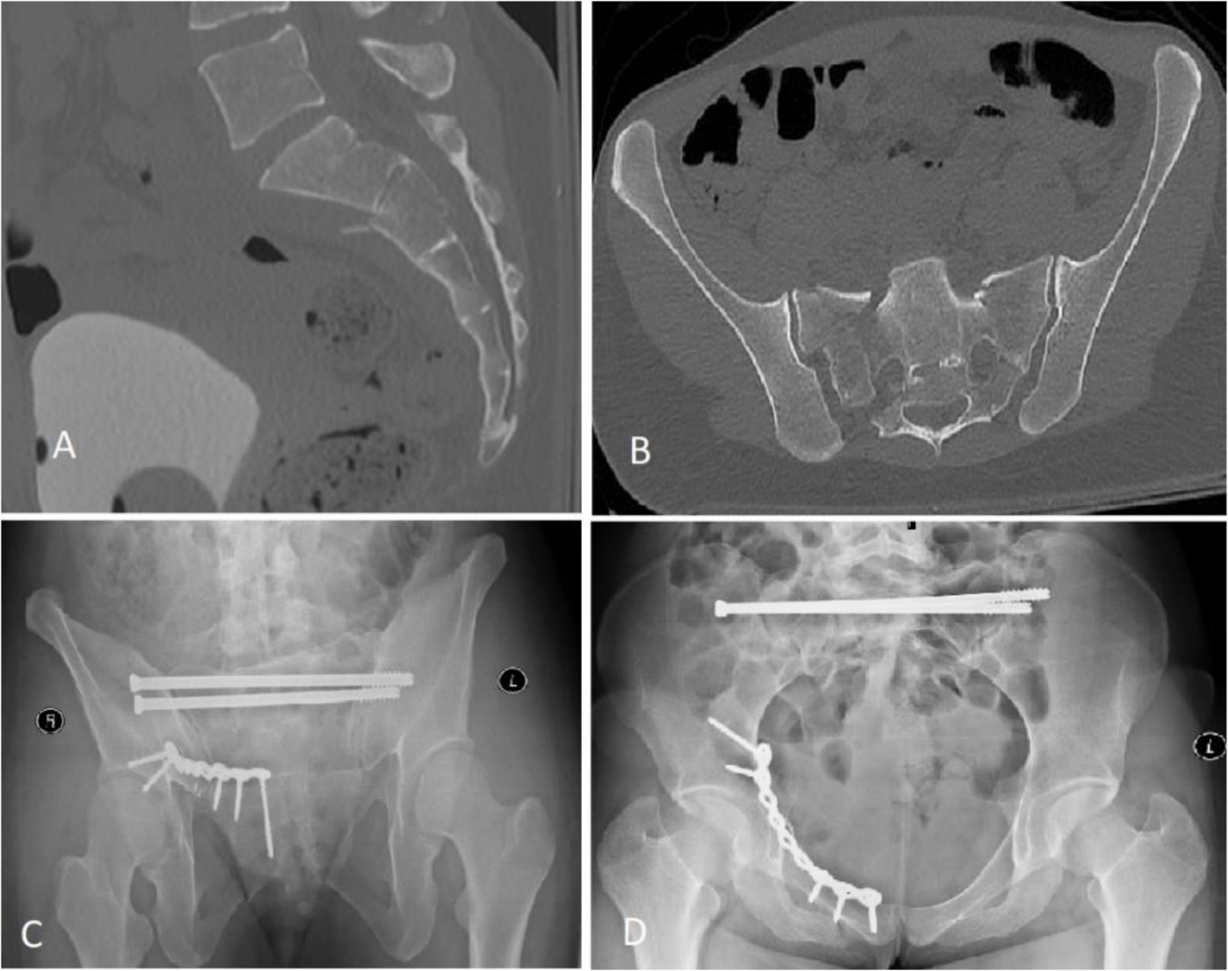
**A-D** Transsacral screw fixation in combination with plate fixation of the anterior pelvic ring

For 3 patients sacral and pubic ramus fractures with minimal displacement were fixed with percutaneous sacroiliac screws alone. For 6 patients with sacral fracture and minimal displacement, pelvic ring stability was achieved with percutaneous transsacral screws in combination with anterior ring fixation by plate or screws (Fig. 2).

**Fig. 3:**
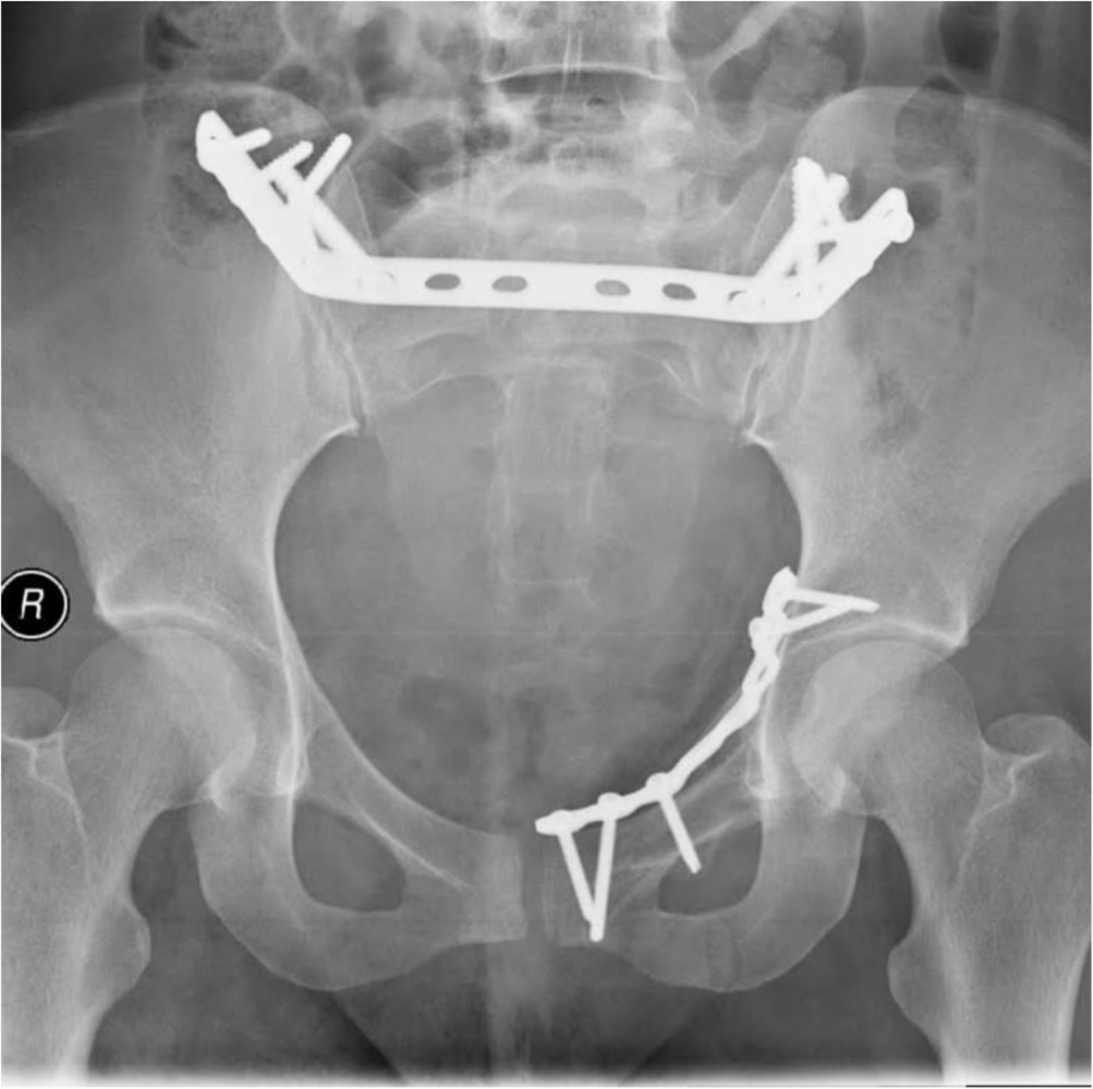
Transsacral plate fixation in combination with plate fixation of the anterior pelvic ring

Transsacral dynamic compression plate (DCP) fixation in combination with anterior pelvic ring fixation by plate was performed in three patients (Fig. 3).

**Fig. 4:**
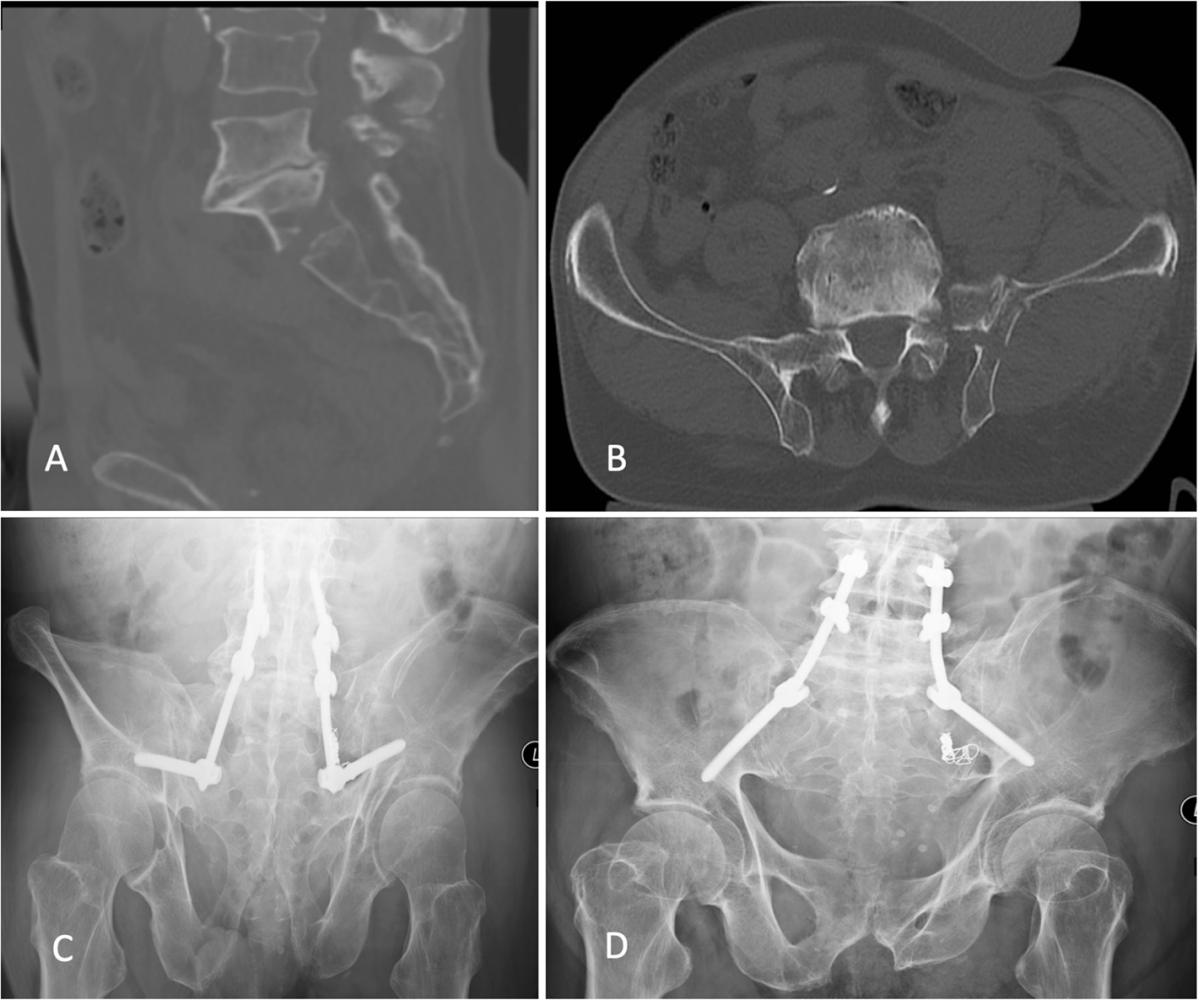
**A-D** Spinopelvic fixation of the U-shaped sacral fracture

Three patients underwent spinopelvic fixation (Fig. 4), of which two were triangular fixation.

Partial weight-bearing was allowed on the next day after surgical stabilization. Full weight bearing would be possible for patients subjected to triangular fixation, however we allowed partial weight-bearing for all of our patients to unify the postoperative course. Only four patients were not able to be mobilized on the next day due to other bone fractures or concomitant injuries. Full weight-bearing was allowed 6–8 weeks after surgery, depending on clinical, radiological signs of fracture healing and associated injuries. Anticoagulants were administered for 4 weeks after injury. On the first day after surgery, analgesia was performed with opioids; and on the second and further days, non-steroidal anti-inflammatory drugs were used.

Statistical analysis was performed using IMB SPSS Statistics v23.0 and figures are presented as mean ± standard deviation. The Mann-Whitney U, Wilcoxon, and Kruskal-Wallis tests were used for the comparison of groups. Differences were considered significant at *p* < 0.05.

## Results

A total of 16 patients were enrolled in the final analysis, 10 (62.5 %) patients were female and 6 (37.5 %) were male. The mean age of the patients was 40.2 ± 17.7 years. All patients worked before their injury. The mean ISS was 19.7 ± 8.5 points. The mean duration of hospitalization was 27.1 ± 19.0 days. The mean time from admission to surgery was 7.8 ± 8.2 days, while the mean duration of surgery was 137.2 ± 57.6 min. More detailed information about the patients, including mechanism of injury, Denis and Roy-Camille classifications, and details of surgery, is presented in Table 1.

**Table 1:** Characteristics of the patients and surgery methods

№	Age	Sex	ISS	Mechanism	Denis	Roy-Camille	Sacral kyphosis	Days in hospital	Days until surgery	Surgery time, min	Fixation method	Pre-trauma Majeed	Majeed after 1 year
1	25	F	18	MVA	3	2	25°	9	2	175	PPF + APF	100	96
2	49	M	29	MVA	3	2	32°	20	13	135	PSF + AEF	99	85
3	28	M	18	HF	2	1	10°	14	2	195	PPF + APF	100	73
4	42	F	18	HF	2	1	22°	19	8	90	PSF + APF	100	83
5	65	F	16	HF	1	2	60°	10	15	60	PSF	69	50
6	28	F	17	HF	3	2	34°	46	3	50	PSF + APF	100	98
7	19	M	18	HF	3	2	60°	36	0	75	PSF + APF	100	59
8	19	F	18	HF	2	1	54°	9	2	175	PSF + APF	100	62
9	24	F	21	MVA	2	1	40°	11	13	120	PSF + ASF	100	100
10	28	M	9	HF	2	2	36°	14	2	105	PPF + APF	100	74
11	62	F	9	MVA	3	1	22°	17	8	80	PSF + APF	88	74
12	61	F	18	HF	2	3	32°	34	4	160	SPF	77	72
13	39	F	34	HF	3	3	50°	52	0	260	SPF	100	44
14	27	F	9	HF	3	3	54°	76	28	175	PSF	100	22
15	66	M	40	HF	1	1	10°	40	22	160	SPF	100	84
16	61	M	24	HF	2	3	7°	27	3	180	PSF + APF	100	62

*MVA *motor vehicle accident, *HF *fall from height, *PPF *plate fixation of the posterior pelvic ring, *AEF *external fixation of the anterior pelvic ring, *APF* plate fixation of the anterior pelvic ring, *PSF* percutaneous screw fixation of the posterior pelvic ring, *ASF* percutaneous screw fixation of the anterior pelvic ring, *SPF* spinopelvic fixation

All patients suffered from an anterior pelvic ring injury and 2 patients (number 7 and 15) had an additional iliac fracture.

A total of 14 patients suffered at least one concomitant injury: 13 (81.3 %) fracture(s) of other bone(s); 7 (43.8 %) neurological deficit, 3 (18.8 %) chest injury; 2 (12.5 %) head injury; and 2 (12.5 %) urogenital tract injury. The direct decompression of the sacral nerve roots was performed for one patient (patient number 13). Other surgeries were performed for 11 (68.8 %) patients. Of this cohort, 7 (43.8 %) patients had at least one comorbidity. Regarding functional outcomes and quality of life indicators, there were no statistically significant differences between types of fracture, concomitant injuries, time to surgery, or complications.

One patient (patient number 15) developed a deep wound infection. The neurological status for the majority of our patients with initial traumatic neurological deficit improved after surgery, however residual paresis of the foot was observed in three patients 1 year after injury (patients 3, 14 and 15). Non–union of the sacral fracture, confirmed clinically and using CT scans, was observed in one patient who underwent percutaneous transsacral fixation with a screw (patient number 7).

Patients filled in the Majeed and SF-36 questionnaires twice. According to the mean Majeed pelvic score results, functional outcomes decreased from excellent before the injury to good 1 year after pelvic fracture. According to the results of SF-36, there was a significant decrease in physical activity, and 1 year after the injury it was worse compared with the normal population, however, no difference in mental status was observed. The results of these surveys on functional outcomes and quality of life are presented in the Table 2.

**Table 2:** Comparison of Majeed pelvic score, SF-36 PCS, and SF-36 MCS scores during the follow-up

	Pre-trauma	One-year follow up	*P* value
Majeed	95.81 ± 9.50 95 % CI [90.75, 100.00]	71.13 ± 20.98 95 % CI [59.94, 82.31]	*P* = 0.001
SF-36 PCS	55.87 ± 8.89 95 % CI [51.13, 60.60]	43.45 ± 9.64 95 % CI [38.31, 48.59]	*P* = 0.003
SF-36 MCS	43.76 ± 12.45 95 % CI [37.13, 50.39]	43.41 ± 7.56 95 % CI [39.38, 47.44]	*P* = 0.501

**Fig. 5:**
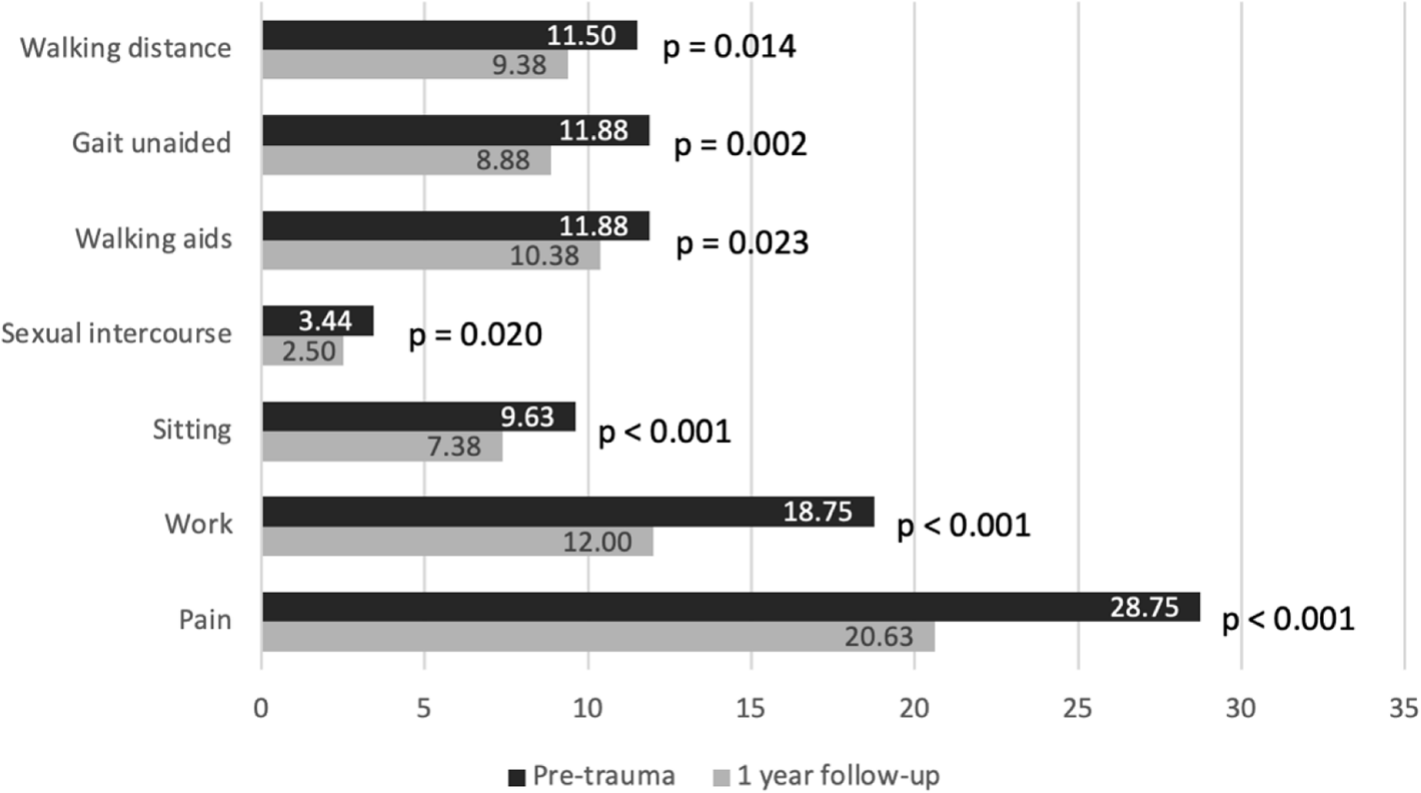
Functional outcomes evaluation according to Majeed pelvic score categories. The differences within a year were statistically significant in all of the categories

**Fig. 6:**
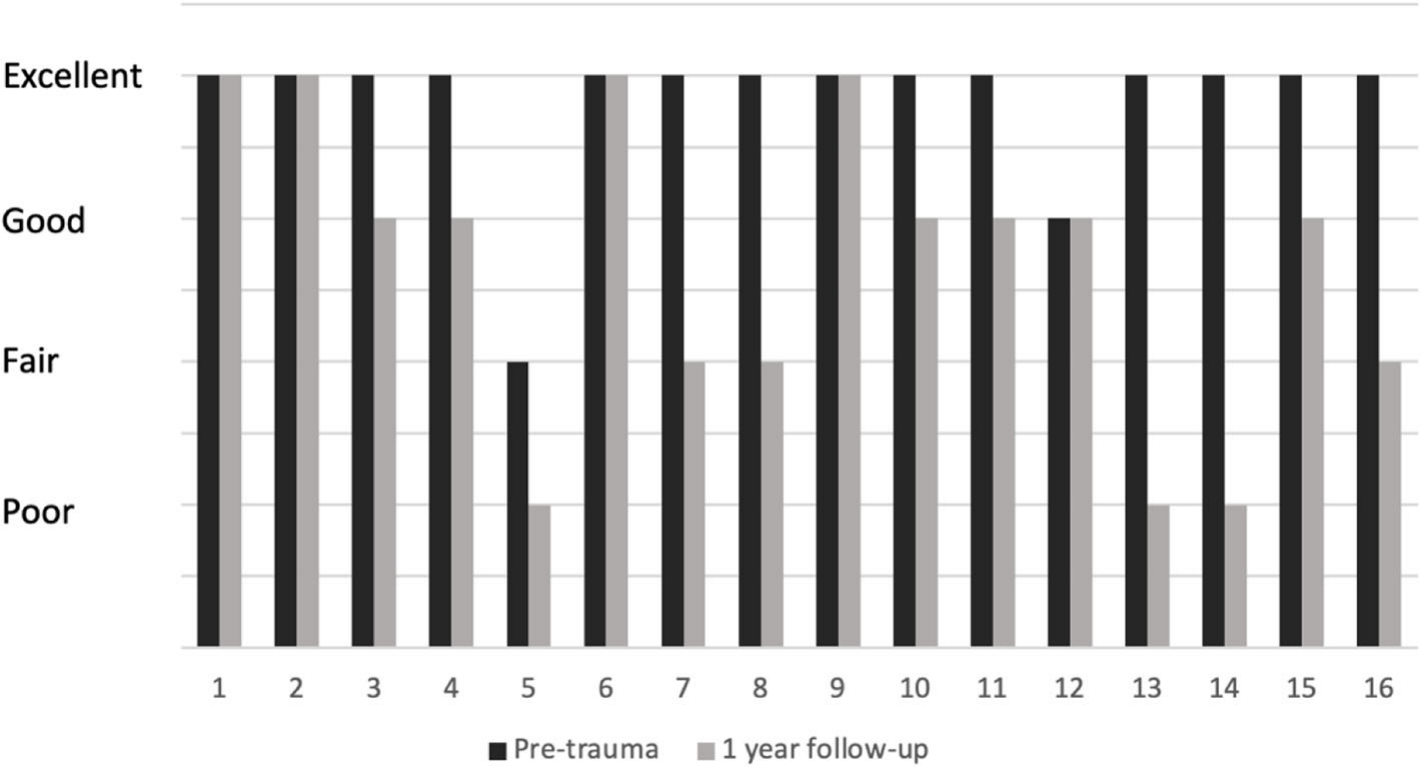
Pre-trauma and 1-year after injury functional outcomes of all 16 patients. A total of 5 (31 %) of the patients achieved pre-traumatic functional outcomes after 1 year

Significant changes were found in all categories of the Majeed pelvic score within a year (Fig. 5). A total of five patients returned to their pre-traumatic functional state after 1 year (Fig. 6), however, we did not find factors influencing better outcomes.

## Discussion

Our study confirms that spinopelvic dissociation is a rare injury—representing only 4 % of all patients with pelvic fractures treated in our centre over a four-year period. The literature describes the mechanism of trauma as a fall from height that first impacts the feet, during which the action of mechanical force causes the dissociation of the spine from the pelvic ring [8, 16]. In our study, all of the patients with spinopelvic dissociation suffered high-energy trauma, with 76.5 % involving a fall from height. According to the data of most authors, the dominant trauma mechanism of spinopelvic dissociation is similar to our “fall from height” [4, 8, 17, 18].

Spinopelvic dissociation severely restricts a patient’s movement. When treating these injuries, the goal is to perform a final fixation as soon as possible that might reduce pain and the use of analgesics, as well as minimizing the risk of deep vein thrombosis, pulmonary thromboembolisms, and the formation of pressure ulcers via the faster mobilization of the patient. However, there is no association described between the timing of final sacral fixation and clinical outcomes [19, 20]. In the literature, this time varies between 4 and 10 days [3, 9, 16]. In our study, final sacral fixation was performed after an average of seven days, after the stabilization of the patient’s condition.

One of the factors influencing short-term and long-term functional outcomes after pelvic fracture is nerve root injury [19, 21]. There is much controversy in the literature regarding the decompression of the nerve roots, and there are no clear indications for decompression in the presence of neurological symptoms in U-shaped sacral fractures [22, 23]. The latest meta-analysis data provided by Bäcker et al. suggests that neurological lesions occur in 68.1 % of patients who experience U-shaped sacral fractures, and 65.1 % of these patients recover after surgical treatment [18]. According to our study, 43.8 % of all fractures were associated with neurological impairment—of which 57.1 % improved after surgery. Lindahl et al. conducted a study of 36 patients with spinopelvic dissociation, 35 of whom had clinical neurological symptoms, and found no association between time to spinopelvic fixation accompanied with decompression and neurological lesion recovery or improved functional outcomes. We agree with the conclusions of the authors—that a more accurate repositioning of the sacral fracture is the key to restoring neurological function [19]. Indirect decompression was performed on the majority of the patients in our study, but a significant discrepancy between functional outcomes, quality of life, and time to surgery was not found. Kepler et al. performed a literature analysis and also found no difference between direct and indirect decompression in terms of neurological recovery [24].

The assessment of functional outcomes and the quality of life of patients with spinopelvic dissociation is complicated as these fractures are very rare, and the small cohort of patients that suffer them often experience many concomitant injuries that also affect outcomes. Gribnau et al. retrospectively examined quality of life and pelvic function in eight patients using the EQ-6D questionnaire after the treatment of Denis type III sacral fractures. On average, the follow-up lasted 36 months. Pain, mood disorders, and movement problems were found to be common amongst these patients in the future, but self-care and daily activities were not affected [16]. Ayoub examined 28 patients with spinopelvic dissociation with cauda equina syndrome and satisfactory results were obtained for 67.9 % of the patients [25]. Lindahl et al. found that complete displacement of the transverse sacral fracture and paraplegia or paraparesis were prognostic factors of poor clinical outcomes [19]. Ruatti et al. conducted a study that similarly assessed functional outcomes after U-shaped fractures using Majeed and Iowa questionnaires. A retrospective study was performed, consisting of 20 patients and a mean follow-up of 42 months. Excellent functional results were obtained in 10 patients, very good in 9 patients, and good in 1 patient. The average Majeed score was 86.6 points, and the average Iowa score was 79 points [26]. Compared to our data (with a mean Majeed score of 71.13 points), better results were obtained, but the patients in the Ruatti et al. study had isolated sacral fractures which may have influenced their higher results. Erkan et al. prospectively followed 19 patients who underwent triangular osteosynthesis after spinopelvic dissociation and examined quality of life and functional outcomes after 1 year, using Majeed and EQ-6D questionnaires. The data of functional outcomes provided by the authors was better than ours: after 1 year, 12 (63.2 %) of their patients reported excellent results, 5 (26.3 %) good, and 2 (10.5 %) fair. In our study, after 1 year, 4 (25 %) patients reported excellent results, 6 (37.5 %) good, 3 (18.7 %) fair, and 3 (18.7 %) poor in response to our Majeed questionnaire [11]. He et al. prospectively examined the outcomes of 41 patients with U-shaped sacral fractures. The mean duration of the follow-up was 4.1 years after surgery. The authors concluded that the impairments improved during the first year after the injury, however the neurological status improved only for one third of the patients. These results are consistent with the results of our research. Nevertheless, He et al. emphasised that neurological impairment appeared to persist if not resolved within a year [27]. Several other authors who applied triangular osteosynthesis also attained better results than ours. Tian et al. reported that functional outcomes evaluated with a Majeed questionnaire for 12 (66 %) of their patients were excellent, 4 (22 %) good, and 2 (11 %) fair [28]. Similarly, Hu et al. reported 13 patients (59 %) with excellent functional outcomes, 6 (27 %) good, 2 (9 %) fair, and 1 (4 %) poor [29]. The internal fixations performed on our patients were mainly percutaneous and minimally invasive, suggesting that the fixation was not sufficiently stable and contributed to poorer functional outcomes. Such a suggestion cannot be conclusive, however, as this would require a randomized comparative study. As such, this study could serve as an incentive for further randomized comparative study. In addition, we believe that concomitant injuries, complications and generally more severe initial injury made the biggest impact on the suboptimal performance in the outcomes for some of our patients because no significant differences in the therapy of these patients were observed.

Erkan et al. points out that the quality of life studied with the EQ-6D questionnaire is influenced by pain and mood [11]. The SF-36 was used in our study, and we did not find any significant differences between MCS before trauma and after 1 year. However, there was a noticeable difference in PCS, which decreased significantly after 1 year (Table 2). This result is in agreement with the conclusion of Gribnau et al. that most patients report mobility problems after the surgical treatment of U-shaped sacral fractures [16].

There are several limitations to this study. Firstly, this is a single-centre study. Secondly, no formal surgery protocol for spinopelvic dissociation was used. Therefore, the surgery technique and the choice of fixation was based on the preference of the attending surgeon. Due to the heterogeneity of associated injuries and small sample size, it was not possible to determine the effect of other injuries on the quality of life and functional outcomes. Nevertheless, knowing the rarity of this trauma, our article is one of the few that presents functional outcomes and quality of life after spinopelvic dissociation and our findings may provide a reference for future studies.

## Conclusions

According to the data of our study, patients who have experienced spinopelvic dissociation have a significant decrease in functional outcomes, which affects physical activity at work and in daily life. Only one-third of patients achieved pre-traumatic functional outcomes after 1 year.

## Data Availability

The datasets used and analyzed during the current study are available from the corresponding author on reasonable request.
